# Cytoskeletal prestress: The cellular hallmark in mechanobiology and mechanomedicine

**DOI:** 10.1002/cm.21658

**Published:** 2021-05-01

**Authors:** Farhan Chowdhury, Bo Huang, Ning Wang

**Affiliations:** ^1^ Department of Mechanical Engineering and Energy Processes Southern Illinois University Carbondale Carbondale Illinois USA; ^2^ Department of Immunology, Institute of Basic Medical Sciences & State Key Laboratory of Medical Molecular Biology Chinese Academy of Medical Sciences and Peking Union Medical College Beijing China; ^3^ Department of Mechanical Science and Engineering University of Illinois at Urbana‐Champaign Urbana Illinois USA

**Keywords:** cell softness, extracellular vesicles, immune cells, stem cells, substrate stiffness, tumor metastasis

## Abstract

Increasing evidence demonstrates that mechanical forces, in addition to soluble molecules, impact cell and tissue functions in physiology and diseases. How living cells integrate mechanical signals to perform appropriate biological functions is an area of intense investigation. Here, we review the evidence of the central role of cytoskeletal prestress in mechanotransduction and mechanobiology. Elevating cytoskeletal prestress increases cell stiffness and reinforces cell stiffening, facilitates long‐range cytoplasmic mechanotransduction via integrins, enables direct chromatin stretching and rapid gene expression, spurs embryonic development and stem cell differentiation, and boosts immune cell activation and killing of tumor cells whereas lowering cytoskeletal prestress maintains embryonic stem cell pluripotency, promotes tumorigenesis and metastasis of stem cell‐like malignant tumor‐repopulating cells, and elevates drug delivery efficiency of soft‐tumor‐cell‐derived microparticles. The overwhelming evidence suggests that the cytoskeletal prestress is the governing principle and the cellular hallmark in mechanobiology. The application of mechanobiology to medicine (mechanomedicine) is rapidly emerging and may help advance human health and improve diagnostics, treatment, and therapeutics of diseases.

## 1 BACKGROUND AND INTRODUCTION

Force, body structure and mechanics, and movements in animals and humans have been recognized for centuries by the giants like Aristotle, Archimedes, da Vinci, Galileo, Newton, and Borelli (Fung, 1981). In the second half of the 19th century, Julies Wolff postulated Wolff's law that the bone remodels itself over time to resist mechanical loading (Frost, 2004). Early in the 20th century, D'Arcy Thompson proposed that physical laws and mechanics play critical roles in the evolution of living organisms' structure and form (Thompson, 1917). In the 1950s and 1960s, at the level of the human body, research efforts were initiated by a few pioneers (YC Fung, Jere Mead, and Al Burstein) to understand biomechanical functions in systems like the respiratory system (Avery & Mead, 1959; Mead, 1961; Mead, Lindgren, & Gaensler, 1955), the cardiovascular system (Fung, 1966a, 1966b; Fung & Sobin, 1969), and the musculoskeletal system (Burstein & Frankel, 1968), which are all known to experience forces and/or deformation in the body. These pioneering works and later research works in these areas (Macklem, 1998; Taylor & Draney, 2004; Woo & Kim, 2012; Wootton & Ku, 1999) have led to effective therapeutics in medicine (including sports medicine) such as the development of delivery of artificial surfactant to premature babies to reduce lung surface tension, the use of stents to open blood vessel obstruction, and orthopedic implants/prostheses to help patients. On the other hand, at the level of biological molecules, it is well‐known that forces associated with covalent and noncovalent bonds are critical in structure, specificity, syntheses, and functions of DNA, RNA, proteins, lipids, and polysaccharides (Lodish, Berk, Matsudaira, & Kaiser, 2003). However, at the level of individual living cells, it is only during the last few decades that it has become increasingly evident that forces influence gene expression, protein synthesis, proliferation and apoptosis, embryonic development, cell fate decisions, migration and invasion in physiology and diseases. Here, we review the central role of cytoskeletal prestress (pre‐existing tensile stress in the cytoskeleton) in cellular mechanotransduction (conversion of mechanical signals into biochemical signals or gene expression) and mechanobiology (a study of mechanical basis of biology). Then we highlight recent advances in the emerging interdisciplinary area of mechanomedicine (mechanobiology‐based medicine) (Naruse, 2018; Wang, 2009, 2017) and discuss challenges and opportunities.

## 2 THE DISCOVERY OF INTEGRIN MECHANOSENSORS

For many years, researchers had always used charged culture dishes to seed cells, believing that cells attaching to their environment nonspecifically. A major breakthrough came when it was shown that a small fragment in the fibronectin extracellular matrix (ECM) protein promotes cell adhesion (Pierschbacher, Ruoslahti, Sundelin, Lind, & Peterson, 1982). Subsequent studies with synthetic peptides showed that it is a repeating tripeptide sequence RGD that promotes cell adhesion (Pierschbacher & Ruoslahti, 1984). Identifying the minimal cell adhesion sequence of fibronectin was very instrumental toward our understanding of cell adhesion. The other key to the puzzle is to identify the transmembrane protein itself. In this regard, a few monoclonal antibodies incidentally blocked cell adhesion to matrix protein‐coated tissue culture dishes (Damsky, Knudsen, Bradley, Buck, & Horwitz, 1985; Knudsen, Horwitz, & Buck, 1985). With these monoclonal antibodies, a cell surface protein that is responsible for adhesion to fibronectin was identified and cloned (Gardner & Hynes, 1985; Tamkun et al., 1986). A few years before that, researchers worked on surface antigens on T cells (Sanchez‐Madrid et al., 1982). Around the same time, cell surface adhesion proteins in *Drosophila* (Wilcox, Brown, Piovant, Smith, & White, 1984), immune cells (Hemler, Jacobson, Brenner, Mann, & Strominger, 1985), lymphoid and myeloid cells (Springer, Miller, & Anderson, 1986), and platelets (Parise & Phillips, 1985) were identified. It later became increasingly clear that all these studies converged and pointed toward the discovery of the same class of cell adhesion receptors although they were named differently at the time. It was not until 1986 that the term “integrin” was chosen to represent this class of adhesion‐related integral membrane glycoprotein (Tamkun et al., 1986).

**FIGURE 1:**
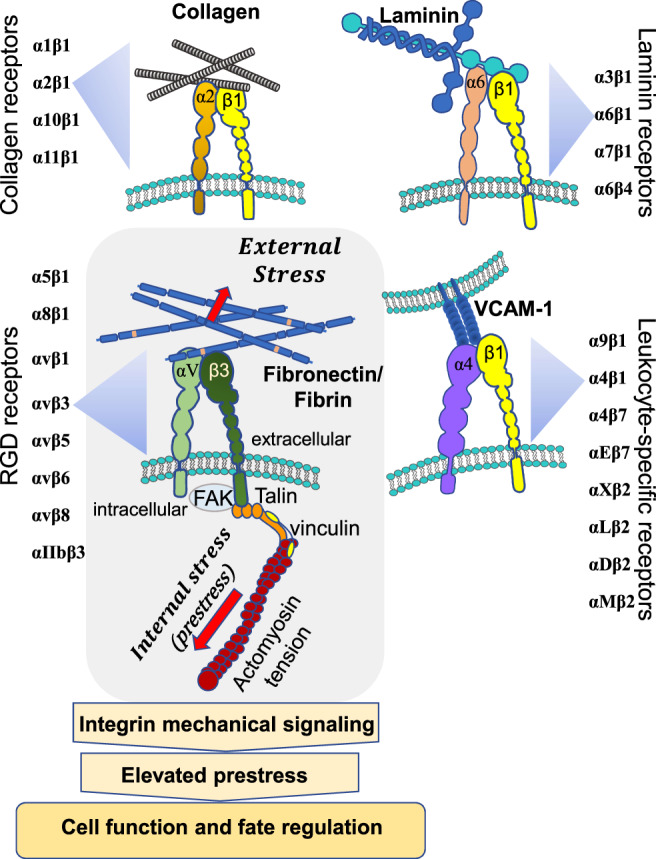
The heterodimeric integrin superfamily and their corresponding ligand binding components. In mammals, 18 α and 8 β subunits give rise to 24 different αβ integrin receptor combinations. β1 subunits are the most commonly found. Integrin activation leads to the accumulation of cytoplasmic FA (focal adhesion) proteins connecting to actomyosin (myosin II and filamentous actin) and elevation of the cytoskeletal prestress that all living cells generate. The cytoskeletal prestress is balanced at other anchoring sites. FAK: Focal adhesion kinase

Significant advances in integrin biology have followed those early studies in the 1980s. It is shown that mammalian genomes encode 18 α and 8 β subunit genes, giving rise to 24 different αβ subunit combinations (Humphries, 2006; Hynes, 2002). Among these, the β1 subunit is one of the major subunits that appear in 12 different types of integrins (Figure 1), possibly causing occasional promiscuity for ECM binding (Hynes & Naba, 2011). Nevertheless, the RGD‐sequence has remained the center of integrin research as all five α_V_ integrins, two of the β1 integrins (α5β1 and α8β1), and αIIbβ3 integrins share the ability to bind to the RGD sequence. Furthermore, the active and inactive structures of integrins are also identified (Takagi, 2003). When an integrin is inactive, the ectodomain remains in bent conformation while the hybrid domain assumes a closed configuration thus preventing actin cytoskeleton binding via cytoplasmic focal adhesion (FA) proteins. Upon binding to the fibronectin domain‐containing RGD sequence, integrins are activated with ectodomains extended and swing opening of the hybrid domains by ~7 nm (Takagi, 2003), an opening that is assumed to be enough for linking actin cytoskeleton via the cytoplasmic FA proteins. However, the details of these processes are still not well understood at this time and remain an active area of research. In the late 1980s and early 1990s, most research had been focused on the biochemical signaling cascades downstream from integrins upon cell‐matrix adhesion until it was discovered that integrins and FAs mediate mechanical force transmission to the actin cytoskeleton (Wang, Butler, & Ingber, 1993). Cells exhibit force‐dependent stiffening response by integrins but not by nonspecific scavenger receptors. Such a demonstration shows that integrins act as mechanosensors. In the following years, integrins are found to be responsible for outside‐in and inside‐out bidirectional force signaling and stiffening (Balaban et al., 2001; Choquet, Felsenfeld, & Sheetz, 1997; Pelham & Wang, 1997) (Figure 1). Although the detailed mechanisms of mechanosensitivity of different integrin subtypes remain unclear, receptors of fibronectin (α5β1) and type 1 collagen (α2β1) are shown to distinctly regulate force‐induced activation of FAK (focal adhesion kinase), an enzyme that binds to the tail of integrin (Seong et al., 2013). A recent study reveals that nanoscale spacing influences different drug sensitivity of αvβ3 and α5β1 in cancer cells (Young et al., 2020). Functional consequences of integrin gene mutations in mice have been reviewed in an earlier article (Bouvard et al., 2001), but no mechanosensitivity tests in cells from the mice are performed in those early studies. Mice with inactivated β1 integrin exhibit a chondrodysplasia phenotype. β1‐deficient chondrocytes from these mice have an abnormal shape and fail to arrange into columns in the growth plate, due to a lack of motility, which is caused by a loss of adhesion to type II collagen, reduced binding to and impaired spreading on fibronectin, and an abnormal F‐actin organization, suggesting a defective mechanosensitivity in these cells (Aszodi, Hunziker, Brakebusch, & Fässler, 2003). In contrast, α10‐null mice only exhibit a mild chondrodysplasia with moderate dysfunction of growth plate chondrocytes, possibly due to compensation by α2β1 (Bengtsson et al., 2005). α7β1‐integrin is increased in skeletal muscle in humans and mice lacking dystrophin to compensate for the lack of the transmembrane adhesion (Hodges et al., 1997). In addition, mice overexpressing α7β1 integrin show resistance to exercise‐induced muscle damage, suggesting α7β1 provides protection against hyper‐force‐transduction (Boppart, Burkin, & Kaufman, 2006). A recent review has discussed the latest advances in force‐induced integrin signaling and skeletal muscle hypertrophy (Boppart & Mahmassani, 2019). It is clear that more concrete work is needed in the future to determine alterations in cellular mechanosensitivity in various cell types in integrin‐null mice.

When stresses are applied at the plasma membrane, membrane proteins can be distorted or deformed. It is known that Piezo‐1 and stretch‐activated ion channels play important roles in endothelial cells' and other cells' responses to fluid shear stress or stretches (Arishe, Ebeigbe, & Webb, 2020; Gerhold & Schwartz, 2016; Lansman, Hallam, & Rink, 1987; Nonomura et al., 2018); cytoskeletal prestress could regulate opening and activation of these plasma membrane proteins by modulating membrane tension. Importantly, the revelation that integrin‐mediated mechanotransduction in response to shear stress (Jalali et al., 2001; Liu et al., 2002) and Rho‐dependent cytoskeletal remodeling in the endothelial cells in response to stretch (Kaunas, Nguyen, Usami, & Chien, 2005) regulate endothelial homeostasis suggests that cytoskeletal prestress is important in these cellular responses. The topic of endothelial cell mechanotransduction and homeostasis has been reviewed elsewhere (Chien, 2007).

It is now well established that integrins mediate mechanical signaling, recruit cytoplasmic FA proteins (Geiger & Bershadsky, 2002; Geiger, Spatz, & Bershadsky, 2009), and propagate mechanical stresses along the cytoskeleton to alter functions of other cytoplasmic proteins and even nuclear proteins and cellular responses in general. It has been shown that not only relatively stable structures like FAs (Smilenov, Mikhailov, Pelham, Marcantonio, & Gundersen, 1999) and fibrillary adhesions (Barber‐Pérez et al., 2020) mediate force transmission across the cell surface, dynamic integrin‐containing structures like podosomes (Collin et al., 2008), invadopodia (Alexander et al., 2008), and focal complexes (Beningo, Dembo, Kaverina, Small, & Wang, 2001) are also capable of transmitting bi‐directional forces across the plasma membrane. Downstream of integrins, the intracellular FA proteins like talin and vinculin have been investigated for their mechanosensing ability. Tyrosine phosphorylation in the cytoplasm downstream from integrin and cytoskeletal mechanosensing is shown to be one of the early events of mechanotransduction (Sawada et al., 2006). It has been shown that stretching of purified single talin rods activates vinculin binding and subsequent binding to the actin cytoskeleton (del Rio et al., 2009). This notion is also supported by an investigation where the spacing between integrin binding sites is precisely controlled demonstrating that FA formation is not possible when integrin spacing is more than ~60 nm (Arnold et al., 2004). Taking the cues from the discovery of integrins as mechanosensors, one might expect that any transmembrane adhesion molecule whose cytoplasmic tail(s) has direct or indirect structural linkages with filamentous actin (F‐actin) should be a candidate for mechanosensing. Indeed other transmembrane cell–cell adhesion molecules have been shown to act as mechanosensors: E‐selectins (Yoshida et al., 1996), platelet‐endothelial cell adhesion molecule‐1 (PECAM‐1) (Tzima et al., 2005), and E‐cadherins (le Duc et al., 2010). These molecules play different roles from integrins in regulating cell functions. For example, E‐cadherins are important in mediating cell–cell mechanical signaling and tissue integrity and dynamics (Lecuit & Yap, 2015). In this article we focus mainly on mechanotransduction via integrins.

## 3 CYTOSKELETAL PRESTRESS IN MECHANOTRANSDUCTION

### 3.1 Quantifying cell stiffness

To study mechanotransduction via integrins, one must first apply a mechanical load to a cell. For any applied force, the contact area between the force probe and the cell is critical and should be defined. For example, for a given force magnitude, the smaller the contact area, the higher the impact of the force. It is the force per unit area, that is, the stress, but not the force per se, that a living cell responds to. Since the force has a unit of Newton, the stress must have a unit of Newton per square meter or Pascal (Pa) (Table 1). The applied stress causes distortion of the plasma membrane, the cytoplasm including the cytoskeleton, and even the nucleus, indicating that these structures are deformed or strained by the applied stress (Table 1). Strain is defined as the deformation resulting from an applied stress or the ratio of the change in length to the original length and is thus dimensionless (Table 1). To characterize the ability to resist deformation in response to an applied stress, a general term “stiffness” is used, which is defined as the ratio of stress to strain and therefore has the same unit as stress (Table 1). A specific term “modulus” is often used to replace stiffness and modulus can be subdivided to reflect the response to different modes of stress. For example, Young's modulus refers to a response to a tensile (stretching) stress, a compressive modulus refers to a response to a compressive stress, and shear modulus refers to a response to a shear stress (e.g., blood flow induced shear stress). An elastic (or storage) modulus refers to the ability to elastically store stress and a dissipative (or loss) modulus refers to the ability to dissipate stress (Table 1). In this article, we use the generic term stiffness in most cases. Stiffness is an intrinsic variable of materials including biological materials. The stiffness of normal human tissues ranges from ~0.1 kPa (1 kPa = 1,000 Pa) in bone marrow to hundreds of MPa (1 MPa = 10^6^ Pa) in bone (Discher, Mooney, & Zandstra, 2009). In some diseases such as arteriosclerosis, aneurysm, or fibrosis, tissue stiffness is perturbed to values well above or below the physiological range in cases such as hardening of arteries (e.g., arteriosclerosis), weakening of blood vessels (e.g., aneurysm), or stiffening of tissues (e.g., fibrosis) as a result of excess fibrous connective tissues.

**TABLE 1:** Definition of technical terminologies

Terms	Definition	Elemental illustrations
Force	The physical quantity, F, when subjected, causes objects such as cells to deform and/or to move. Force has the unit of Newton. Often “force” is used generically to represent a mechanical load.	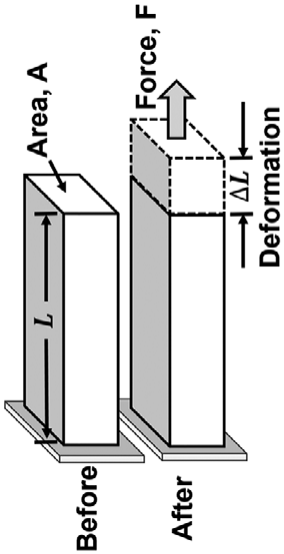
Deformation	The change in geometric length, L, when cells are subjected to forces. The extent of deformation for a given force will depend on the intrinsic stiffness of the material or the cell.	
Stress	A normalized force quantity: The force applied per unit area. $\text{stress}\;\sigma =\frac{F}{A}$. A is the area. The unit is Newton per square meter or Pascal (Pa). It includes both external (applied) stress and internal stress. Please refer to Figure 1. A more generic definition of “stress” is a living organism's response to environmental challenges or external stimuli/events, but we limit the stress discussed in this review to “mechanical stress.”	
Strain	The deformation per original length due to the applied stress. $\varepsilon =\frac{\text{∆}L}{L}$. Strain is dimensionless.	
Stiffness or modulus	The ability to resist deformation in response to applied stress is the stiffness or modulus, E $=\frac{\sigma }{\varepsilon }$. Stiffness or modulus has the same unit as stress, that is, Pa. Depending on the mode of loading, Young's modulus in response to tensile stress (σ), compressive modulus to compressive stress (σ in the opposite direction), or shear modulus to shear stress (τ) can be calculated. The storage and loss moduli represent the elastic (stored) and the dissipated (into heat or other losses) portion of the applied energy. Stiffness is a more generic term.	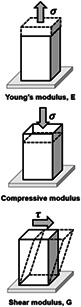
Softness	It is the inverse of stiffness, useful in describing very soft materials.	
Prestress	Existing internal tensile stress. For a living cell, it is generated by myosin‐II mediated actomyosin contractility and called “cytoskeletal prestress.” Please refer to Figure 1 for a detailed illustration.	
Traction	Interfacial stress: Stress at the interface between a cell and the extracellular matrix or a cell and another cell. Traction has the unit of Pa.	
Torque	A twisting force that causes rotation. Torque has the unit of Newton‐meter.	

The importance of cell stiffness to biology has just been emerging in recent years. Looking at the evolution of cell stiffness, one can find that bacteria and archaea are very stiff: they have a stiffness of ~1,000 kPa (Engelhardt, 2007; Francius, Domenech, Mingeot‐Leclercq, & Dufrêne, 2008). On the other hand, while the stiffness value of the very first eukaryotic cell is not known, a single–celled primitive organism protozoan such as an amoeba has a stiffness of only ~0.1 kPa (Reichl et al., 2008). It is now known that metazoan animal cells from pluripotent stem cells to differentiated tissue cells have a stiffness ranging from ~0.1 to ~10 kPa, suggesting that complex multicellular animal cells (especially land animals) stiffen their cytoskeleton to protect their structural integrity from being irreversibly damaged by external and internal mechanical stresses (Chen & Wang, 2018). However, since metazoan animal cells in multicellular organisms need to move and/or to change shape during development and adulthood, it is postulated that it would be energetically too costly and evolutionarily unfavorable for them to stiffen up to be like bacteria, plant cells, or even bone tissues (Chen & Wang, 2018).

While cell stiffness appears to be important for multicellular animal evolution and for embryonic development and cell differentiation from a fertilized egg, quantifying cell stiffness is not a trivial task. A method of micropipette aspiration was developed in the 1950s (Mitchison & Swann, 1954a) and used in sea‐urchin egg modulus determination (Mitchison & Swann, 1954b). This approach has been used to study suspended red blood cell membrane tension and modulus (Evans, Waugh, & Melnik, 1976; Rand & Burton, 1964). For a detailed description of the micropipette aspiration technique, readers are referred to a review article (Hochmuth, 2000). An optical stretcher method has been developed to measure the stiffness of any suspended cells (Guck et al., 2001). Many methods have been developed over the last few decades to study adherent cells' mechanical properties. One such technology is the particle tracking microrheology that is initially applied to measure moduli of cytoskeletal polymers (Apgar et al., 2000; Crocker et al., 2000) and then used to quantify intracellular moduli (Tseng, Kole, & Wirtz, 2002) (for a detailed description of the method, see a review [Wirtz, 2009]). Three other approaches have been used by numerous labs: laser tweezers to trap a particle on the cell surface, developed by Arthur Ashkin in 1970 (Ashkin, 1970) and later used by him to trap living bacteria (Ashkin & Dziedzic, 1987); atomic force microscopy (AFM) to use a cantilever to indent a cell on its surface, developed by Gerd Binnig in 1980s (Binnig, Quate, & Gerber, 1986); and magnetic twisting cytometry (MTC) to use ligand‐coated magnetic beads to stress the cell surface via integrin receptors or other specific receptors with a torque load (Wang et al., 1993) (see Table 1 for torque definition). The MTC method is modified later using optical imaging to detect magnetic bead displacement (Fabry et al., 2001). A magnetic gradient pulling device has been developed (Bausch, Ziemann, Boulbitch, Jacobson, & Sackmann, 1998). A 3D‐MTC that can apply a local stress in any controlled direction has also been developed (Hu, Chen, & Wang, 2004), which allows for integration with confocal microscopy and STED (stimulated emission detection) nanoscopy (Zhang et al., 2017) and can be utilized to apply different modes of stress (complex stress or shear stress) to the same location of the cell (Wei et al., 2020). Cell stiffness measured by MTC has been compared with that by the laser tweezer method (Laurent et al., 2002). A comparison of various methods to measure cell stiffness in the same cell type shows that different methods probe various components of the mechanical properties of the cells but AFM and MTC measure quite similar values of cell stiffness (Wu et al., 2018). The three types of mechanical probes of laser tweezers, AFM, and MTC for measuring cell mechanical properties, each having its own strengths and limitations, together with other approaches, have facilitated the measurements of adherent cell stiffness in normal and diseased cells under various culture conditions.

It turns out that the cytoskeleton but not the plasma membrane is the primary stress‐bearing element and hence the major contributor to cell stiffness (Maniotis, Chen, & Ingber, 1997; Park et al., 2020; Vahabikashi et al., 2019; Walker, Rizzuto, Godin, & Pelling, 2020; Wang et al., 1993; Wang & Ingber, 1994), as long as the cell surface deformation is relatively small. During large deformation, however, especially when the nucleus is substantially deformed, the stiff nucleus contributes to the whole cell stiffness (Dahl, Ribeiro, & Lammerding, 2008; Harada et al., 2014). In the cytoplasm, the cytoskeleton is a network of three major filament systems: actin microfilaments, microtubules, and intermediate filaments, with numerous crosslinking proteins and myosin II molecular motors. Purified cytoskeletal polymers exhibit nonlinear elasticity behaviors (Storm, Pastore, MacKintosh, Lubensky, & Janmey, 2005), quite different from tissue mechanical properties (van Oosten et al., 2019).

### 3.2 Estimating cytoskeletal prestress via tractions

A living cell generates endogenous forces via actomyosin interactions and these forces must be balanced at all times since there is no acceleration of the cell or its cytoplasmic component. These endogenous forces, sometimes also referred to as cytoskeletal tension, exert their overall impact on the 3D cytoskeletal networks as stresses since it is these stresses that generate strains or deformation of the cytoskeleton and other intracellular structures. Because myosin II‐generated stress along the F‐actin is always tensile, it is called cytoskeletal pre‐existing tensile stress (cytoskeletal prestress) before the application of exogenous stresses (Table 1). While it is rather difficult to quantify cytoskeletal prestress directly, it is possible to use measured tractions (interfacial stresses between the cell surface and its substrate) to estimate cytoskeletal prestress. The method of quantifying cellular tractions was first developed in the late 1990s (Dembo & Wang, 1999; Pelham & Wang, 1997) and the evidence of a cultured nonmuscle cell deforming a flexible rubber substrate had been shown much earlier (Harris, Wild, & Stopak, 1980). Several other methods to quantify 2D tractions on 2D substrates have been developed (Balaban et al., 2001; Butler, Tolić‐Nørrelykke, Fabry, & Fredberg, 2002; Legant et al., 2012; Tan et al., 2003; Tolić‐Nørrelykke, Butler, Chen, & Wang, 2002). Both in‐plane and out‐of‐plane tractions can be measured (del Álamo et al., 2013) and this approach is later extended to include simultaneously measuring the Poisson's ratio of the substratum while also determining the cell tractions (Álvarez‐González et al., 2017). 3D tractions in 3D culture (Cóndor, Steinwachs, Mark, García‐Aznar, & Fabry, 2017; Hall et al., 2016; Legant et al., 2010; Maskarinec, Franck, Tirrell, & Ravichandran, 2009; Vorselen et al., 2020) and 3D tractions both in 3D culture and 3D in vivo (Campàs et al., 2013; Mohagheghian et al., 2018) have been developed. Single cell traction mapping has also been extended to monolayer stress microscopy in collective cell migration (Kim et al., 2013; Serrano et al., 2019). A high‐resolution cell mechanical imaging platform is recently developed and it is found that nanoscale stiffness patterns are governed by intracellular prestress (Mandriota et al., 2019). Over the last two decades, significant understandings have been garnered over how cell tractions impact cellular biological functions.

### 3.3 Microfilaments and associated proteins and prestress

**FIGURE 2:**
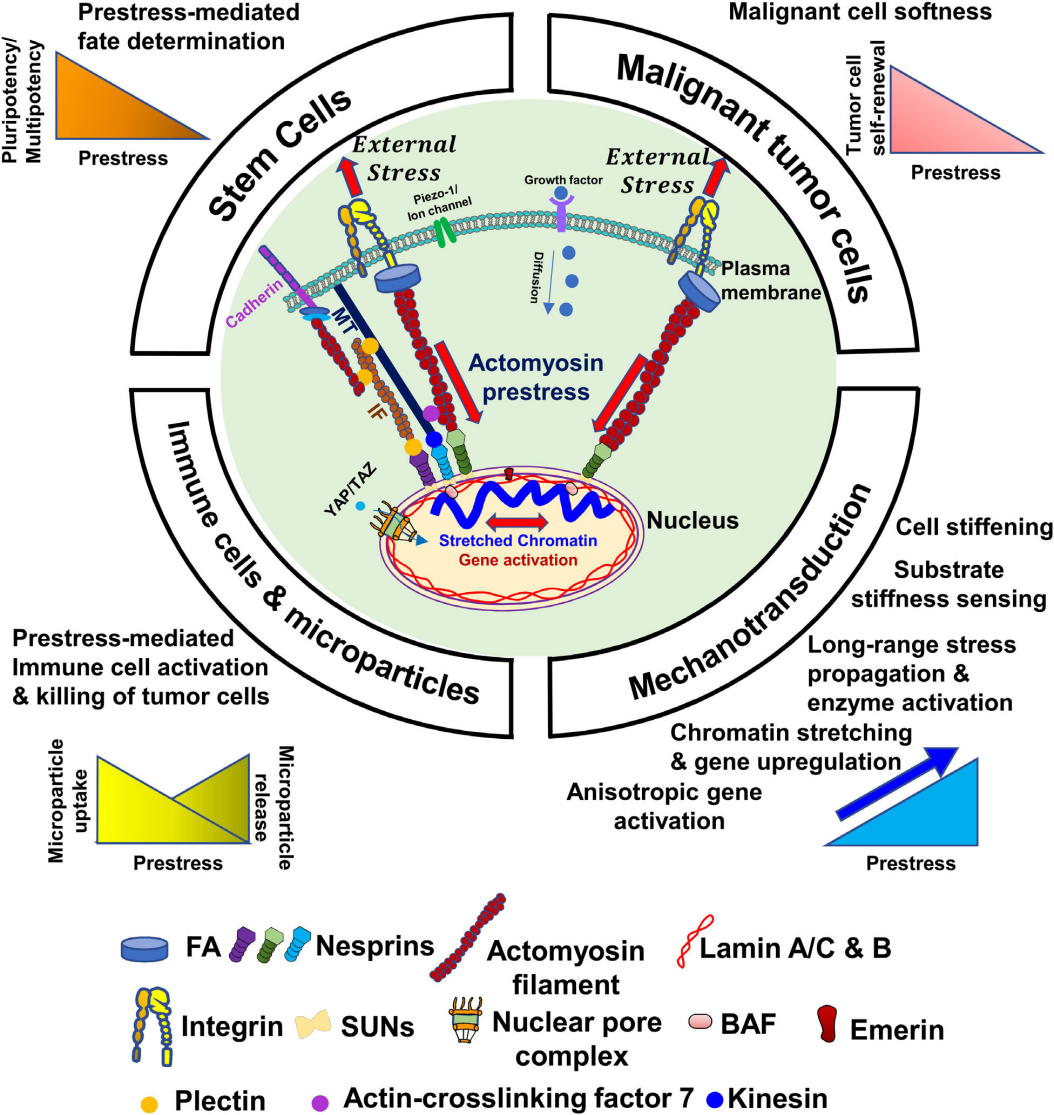
Cytoskeletal prestress is the cellular hallmark in mechanobiology. As one of the primary mechanosensors on the cell surface, integrins mediate cell adhesion and increase actomyosin‐dependent cytoskeletal prestress. Control of cytoskeletal prestress regulates a myriad of cellular functions in addition to embryonic development and cell fate determination. The cytoskeletal prestress is the governing principle and the cellular hallmark. For brevity, a single integrin heterodimer is drawn to illustrate clustered integrins and an actomyosin filament represents a prestressed myosin II‐actin bundle. MT, microtubule. IF, intermediate filament. FA, cytoplasmic focal adhesion proteins. Filamentous actin (F‐actin) interacts with a nesprin. Nesprins (Nesprin‐1 and ‐2 (green), ‐3 (purple) and ‐4 (blue); KASH proteins) and SUNs (Sun 1 and 2) belong to the LINC (linker of nucleoskeleton and cytoskeleton) complex (all not drawn to scale)

Findings with disruption of F‐actin show that actin filament is the most important component of the cytoskeleton to contribute to cell stiffness (Fletcher & Mullins, 2010; Wakatsuki, Schwab, Thompson, & Elson, 2001; Wang, 1998; Wang et al., 1993). The ample evidence that the magnitude of the cell stiffness linearly depends on cytoskeletal prestress in the absence of changes in cell spreading areas indicates that the cytoskeletal prestress is a key determinant of cell stiffness (Hubmayr et al., 1996; Pourati et al., 1998; Cai et al., 1998; Wang & Stamenović, 2000; Wang et al., 2001; N. Wang et al., 2002; Stamenović, Mijailovich, Tolić‐Nørrelykke, Chen, & Wang, 2002). Keeping the cell spreading area constant is important since it is known that the elevation of cell stiffness with substrate stiffness is also associated with cell spreading increases (Yeung et al., 2005) while cell volume reduction as a result of cell spreading can also explain substrate stiffening induced cell stiffening (Guo et al., 2017). The dependence of stiffness on cytoskeletal prestress is later demonstrated in purified actomyosin networks (Gardel et al., 2006) and in various cell types using different methods (Engler et al., 2004; Engler, Sen, Sweeney, & Discher, 2006; Solon, Levental, Sengupta, Georges, & Janmey, 2007). A report reveals that cell stiffness strongly associates with regional tractions and thus cytoskeletal prestress but not with F‐actin density (Park et al., 2010). It is important to note that all living cells, suspended in liquids or attached to substrates, generate cytoskeletal prestress. The cytoskeletal prestress in suspended cells (the intrinsic cytoskeletal prestress) is low and can be elevated dramatically upon cell attachment to the substrate (either ECM or another cell). Rapid cellular stiffening response when the load is applied via integrins depends on the cytoskeletal prestress and not on the mechanosensitive ion channels (Matthews, Overby, Mannix, & Ingber, 2006). These studies paint a picture of cytoskeletal prestress playing a critical role in determining cell stiffness and cell stiffening (Figure 2). Cell stiffening via the cell–cell adhesion molecule cadherin is also dependent on myosin‐II‐driven cytoskeletal prestress (le Duc et al., 2010).

Many actin‐associated proteins can regulate actin polymerization, depolymerization, dynamics, and mechanics (Winder & Ayscough, 2005). One of those proteins is filamin, known as the integrator of F‐actin mechanics and signaling (Stossel et al., 2001). Prestressed F‐actin networks cross‐linked by filamins exhibit similar stiffening responses as living cells (Gardel et al., 2006). Filamin A is shown to be essential for myosin II dependent cell stiffening in living cells (Kasza et al., 2009). External shear stress and myosin‐II‐driven cytoskeletal prestress regulate the binding of β‐integrin tail and FilGAP to filamin A differentially such that strain increases β‐integrin binding to filamin A but causes FilGAP to dissociate from filamin A, providing a direct molecular basis for cellular mechanotransduction at F‐actin (Ehrlicher, Nakamura, Hartwig, Weitz, & Stossel, 2011). Another important actin crosslinker is α‐actinin. It is shown that myosin‐II contractility is required for cytoskeletal coherence (Cai et al., 2010) but the cytoskeletal tension is not sufficient for FA maturation without a stress fiber template which requires α‐actinin to crosslink F‐actin (Oakes, Beckham, Stricker, & Gardel, 2012). A recent study finds that α‐actinin integrates the cytoskeletal prestress spatially to establish an F‐actin network symmetry and FA coherence (Senger et al., 2019). Together it is clear that F‐actin crosslinking proteins such as filamin A and α‐actinin regulate cytoskeletal prestress and cellular mechanical responses.

### 3.4 Intermediate filaments and prestress

Intermediate filaments in metazoan cells constitute two distinct filament systems with one in the nucleus (lamin polymers) and one in the cytoplasm (e.g., vimentin polymers) and are considered to function to support cell shape and to buffer mechanical stress (Herrmann, Bär, Kreplak, Strelkov, & Aebi, 2007). Purified vimentin polymers exhibit stiffening responses at high strains (Storm et al., 2005). In living cells, vimentin intermediate filaments are shown to contribute to cell stiffness and stiffening at large strains (Wang & Stamenović, 2000). Knocking out vimentin diminishes fibroblast tractions (Vahabikashi et al., 2019). Plectin that crosslinks intermediate filaments with microtubules is shown to contribute to cell stiffness, long‐distance stress propagation, and cytoskeletal prestress of living cells (Na et al., 2009). Plectin‐deficient myoblasts, but not plectin‐deficient keratinocytes, exhibit lower mechanical vulnerability upon external stress compared with wild‐type cells, possibly due to lower cytoskeletal prestress in plectin‐deficient myoblasts (Bonakdar et al., 2015). In contrast, vimentin‐null fibroblasts increase nucleus rupture and DNA damage during cell migration in 3D (Patteson et al., 2019). The nuclear lamina, organized by lamin polymers, is shown to protect against nuclear rupture and DNA damage (Cho et al., 2019). In contrast, myosin II inhibition and thus lowering prestress rescue nuclear rupture and partially rescue DNA damage during large deformation as the cells migrate through narrow pores (Xia et al., 2019). Together all these findings suggest that intermediate filaments and their crosslinking proteins contribute to the mechanics of the cell and regulate cellular responses to stresses via prestress.

### 3.5 Microtubules and prestress

Microtubules are made of tubulin proteins and are critical not only for intracellular transport in the cytoplasm but also for chromosome separation during cell division where mechanical stresses must be at play (Forth & Kapoor, 2017). Since the cytoskeletal tensile prestress must be balanced inside the cell by the compressive stress, it is hypothesized that microtubules, a relatively stiff structure, might sustain the compressive stress. Indeed living cell experiments reveal that microtubules balance the tensile prestress (Wang et al., 2002; Wang, Naruse, et al., 2001). Additional theoretical modeling and cell experiments have provided mechanistic insights into how microtubules balance the tensile prestress (Stamenović et al., 2002). Balance of prestress by microtubules and the ECM is controlled by cell spreading (Hu, Chen, & Wang, 2004). The strain magnitude on the FA is shown to be the key factor in regulating prestress balance by the ECM to maintain FA stability and tensional homeostasis (Xu et al., 2020). Lateral reinforcement to microtubules in living cells by surrounding cytoskeletal filaments enhances microtubules' ability to sustain compressive loads before they buckle (Brangwynne et al., 2006). These findings are consistent with a prestressed cell model and are in line with the cellular model of tensegrity that the integrity of structures as being based on a synergy between balanced tension and compression components, first proposed in 1981 (Ingber, 2003; Ingber, 2008; Ingber, Madri, & Jamieson, 1981; Ingber, Wang, & Stamenović, 2014).

### 3.6 Prestress in tissues

Not only cells are prestressed, living tissues such as the lung (Suki & Stamenović, 2011), arteries (Fung & Liu, 1989), and bone (Ascenzi, 1999) are also prestressed, which are important in tissue functions. In a tumor, tumor‐growth‐associated tissue solid stress is generated (Helmlinger, Netti, Lichtenbeld, Melder, & Jain, 1997). Since this tumor solid stress exists before externally applied stress (Nia et al., 2018), this stress can be regarded as tumor tissue prestress, which inhibits tumor spheroid growth (Helmlinger et al., 1997). Therefore, cell and tissue prestress can critically regulate cell and tissue functions in physiology and diseases like cancer.

### 3.7 Cell viscoelasticity and prestress

It is well‐known that a living cell exhibits viscoelastic behaviors but how the cell responds to loading frequency remains elusive for years. A weak power law behavior of living cells has been demonstrated and it is proposed that the cell behaves like a soft glass material (Fabry et al., 2001). These rheological behaviors have been confirmed by numerous studies (Bursac et al., 2005; Fabry et al., 2003; Hoffman, Massiera, Van Citters, & Crocker, 2006; Mandadapu, Govindjee, & Mofrad, 2008; Massiera, Van Citters, Biancaniello, & Crocker, 2007; Semmrich et al., 2007; Trepat et al., 2007). The underlying mechanism of the weak power law is not clear, however, but it has been shown that the nonequilibrium noncovalent bond interactions among proteins can explain this behavior (Chowdhury et al., 2008). It turns out that cytoskeletal prestress also regulates the rheological behaviors of living cells (Stamenović et al., 2004). It is known that the ECM is viscoelastic but how its viscoelasticity impacts cellular functions has been elusive until a report showed that ECM viscoelasticity can impact adult stem cell spreading, proliferation, and differentiation (Chaudhuri et al., 2016).

### 3.8 Prestress in cell spreading, cytokinesis, and migration

For an adherent cell, that cell proliferation is closely associated with cell spreading area was first observed in 1978 (Folkman & Moscona, 1978) and this relationship was investigated further by controlling the density of matrix proteins in cell spreading and proliferation (Ingber, 1990; Ingber & Folkman, 1989). In the late 1990s, it was shown that it was the cell spreading area and not the number of integrins that regulated DNA synthesis (Chen, Mrksich, Huang, Whitesides, & Ingber, 1997). It is then determined that cytoskeletal prestress is the underlying mechanism responsible for cell spreading induced DNA synthesis in single cells and cell monolayers (Nelson et al., 2005). Cytoskeletal prestress is known to regulate growth‐factor‐induced cell cycle entry (Huang, Chen, & Ingber, 1998). Cytoskeletal prestress is also known to regulate cytokinesis, cell division, and cell migration; for reviews in these cell functions, readers are suggested to read these articles (Roubinet, Tran, & Piel, 2012; West‐Foyle & Robinson, 2012).

### 3.9 Prestress in long‐distance cytoplasmic mechanotransduction

For years, a prevailing view in the field is that the greatest force impact on a cell is on the peripheral contacts of the cell and the forces must be dissipated quickly from the local impact (Vogel & Sheetz, 2006). This view is consistent with St. Venant's principle that a local force only causes a local deformation in a homogenous elastic material (Love, 1927). However, if the local load is concentrated inside the cell, then it is likely that the applied stress can propagate much further in the cytoskeleton and exerts long‐distance deformation at sites far from the local stress. Using GFP‐mitochondria as markers of cytoplasmic deformation, it was discovered that intracellular strains and stresses are concentrated at sites tens of micrometers away from the local magnetic bead stress (Hu et al., 2003; Hu et al., 2004), representing a major departure from the then‐prevailing view and the St. Venant's principle. Additional experiments revealed that stiff stress fibers regulate long‐distance stress propagation by concentrating stress (stress focusing) and cytoskeletal prestress is critical in this process (Hu et al., 2003; Hu, Eberhard, et al., 2004). Using a laser nanoscissor to cut single stress fibers in a living cell, it is revealed that stress fibers carry tensile prestress which is important in determining cell shape, cytoskeletal organization, and ECM mechanics (Kumar et al., 2006). Theoretical analyses of a single stiff stress fiber embedded in a soft cytoskeletal network can predict this long‐range stress propagation behavior (Wang & Suo, 2005). Using a fluorescence resonance energy transfer (FRET) based Src activation biosensor (Wang et al., 2005), it is found that cytoplasmic Src enzyme, anchored on the endosome membrane connected to the microtubules, can be rapidly (~100–300 ms) and directly (without intermediate biochemical signaling cascades) activated by a local stress applied via an RGD‐coated magnetic bead, tens of micrometers away from the bead location (Na et al., 2008). The stress‐induced Src activation was more than 40‐fold faster than the soluble growth factor such as the platelet‐derived growth factor (PDGF)‐induced Src activation (Na et al., 2008). Similar stress‐induced enzyme activation of Rac1 at remote sites from the local stress is shown. Remarkably, unlike PDGF‐induced Rac1 activation that depends on prior Src activation, a local stress can directly activate Rac1 in Src‐null cells (Poh et al., 2009). Decreasing cytoskeletal prestress can inhibit long‐distance activation of Src or Rac1 (Figure 2). All these data suggest that cytoskeletal prestress is critical in regulating long‐range cytoplasmic mechanotransduction where stress concentration or stress focusing is essential. The importance of stress concentration at the FAs and at other sites in the cytoskeleton to facilitate long distance stress propagation has often been underappreciated. It is unlikely that membrane tension mediates the long‐distance cytoplasmic mechanotransduction since the plasma membrane is too soft to propagate local stresses to distances greater than 1 μm in the membrane (Shi, Graber, Baumgart, Stone, & Cohen, 2018). Intercellular long‐range force transmission has also been demonstrated during collective cell migration (Sunyer et al., 2016). In addition, long‐range force transmission is found in fibrous matrices via tension‐aligned fibers (Wang, Abhilash, Chen, Wells, & Shenoy, 2014). Rapid force sensing and strengthening (<0.5 s) by single integrins after they engage matrix proteins has also been demonstrated (Strohmeyer, Bharadwaj, Costell, Fässler, & Müller, 2017), consistent with the rapid mechanical force signaling pathway of force transmission from ECM to integrins and to the cytoskeleton. Let us now speculate about the origin of rapid mechanotransduction. In general, cells inside a human body experience three types of signals: chemical, mechanical, and electrical. Propagation of electrical signals generated as a result of action potential by neurons is fastest, followed by propagation of mechanical signals, while the propagation of chemical signals that depend on diffusion or blood/lymphatics flow is the slowest. It is known that primitive single cell organisms that can be as large as several hundred micrometers do not have neurons and thus they must depend on rapid mechanical signal propagation for rapid sensing and response for survival such as catching a prey (Coyle, Flaum, Li, Krishnamurthy, & Prakash, 2019) or avoiding a physical danger of micrometer‐sized particles (Dexter, Prabakaran, & Gunawardena, 2019). It is possible this trait of rapid mechanotransduction has been conserved for millions of years over the course of evolution. Next, we cover the role of cytoskeletal prestress in nuclear mechanotransduction.

### 3.10 Prestress in nuclear mechanotransduction

The nucleus is the largest organelle in the cell and how transcription is regulated is an enigma facing the field of cell biology. While much has been learned on growth factors or cytokines‐induced transcription activation, little is known on how mechanical loads impact nuclear structure and function. Recently it is demonstrated that the nucleus itself acts as a mechanosensor to trigger cellular responses when its membrane is deformed by force‐induced compression in 3D dense matrices (Lomakin et al., 2020). The work in nuclear mechanics research goes back to three decades ago. An early study has revealed that a stiff nucleus can be directly deformed by a fibronectin‐coated micropipette locally deforming the cell surface by 10–20 μm (Maniotis et al., 1997). However, since the magnitude of cell deformation is comparable to the cell diameter, it is not clear if the micropipette deformation is too large to be physiologically relevant. Using an RGD‐coated magnetic bead to apply a physiologically‐relevant local stress of 10–20 Pa, it is shown that the nucleolus inside the nucleus can be directly deformed (Hu, Chen, Butler, & Wang, 2005). Micropipette aspiration of embryonic stem cells and adult stem cells shows that due to lack of lamin A/C their nuclei are much softer than those of differentiated epithelial cells (Pajerowski, Dahl, Zhong, Sammak, & Discher, 2007). In the early 2000s, the proteins of the LINC (linker of nucleoskeleton and cytoskeleton) complex, responsible for linking the cytoskeleton in the cytoplasm to nuclear lamins (Lamin A/C and Lamin B), have been identified (N. Wang, Tytell, & Ingber, 2009; Kirby & Lammerding, 2018), making it possible to study the molecular details of stress‐induced nuclear mechanotransduction.

Numerous studies have shown that nuclear mechanics plays an important role in nuclear structures and function, nuclear lamins behave like mechanosensors, and the nucleus is a prestressed structure (Alisafaei, Jokhun, Shivashankar, & Shenoy, 2019; Banerjee, Bhattacharya, & Shivashankar, 2006; Banigan, Stephens, & Marko, 2017; Chambliss et al., 2013; Guilluy et al., 2014; Ho, Jaalouk, Vartiainen, & Lammerding, 2013; Irianto, Pfeifer, Ivanovska, Swift, & Discher, 2016; Jain, Iyer, Kumar, & Shivashankar, 2013; Kim et al., 2017; Kim, Hah, & Wirtz, 2018; Kim & Wirtz, 2015; Kirby & Lammerding, 2018; Lammerding et al., 2006; Lee et al., 2007; Mazumder, Roopa, Basu, Mahadevan, & Shivashankar, 2008; Pajerowski et al., 2007; Shin et al., 2013; Shin & Discher, 2015; Swift et al., 2013; Swift & Discher, 2014). However, for a mechanical load applied to the cell surface, how the load is transduced into gene transcription has remained unclear for years. One model starts with the FA activation, followed by cytoplasmic biochemical cascades such as YAP/TAZ (Dupont et al., 2011; Elosegui‐Artola et al., 2017; Totaro, Panciera, & Piccolo, 2018) or other molecules like Twist1 (Wei et al., 2015) or MKL1 (Ho et al., 2013), translocating into the nucleus for transcription factors to bind to the chromatin to activate genes. Alternatively, the applied stress at the cell surface via integrins might directly deform the chromatin to activate genes. Using a GFP labeled chromatin domain of transgene DHFR (*dihydrofolate reductase*) developed and reported in a previous study (Hu, Kireev, Plutz, Ashourian, & Belmont, 2009), it is demonstrated that the chromatin can be directly stretched and the extent of gene upregulation is tightly associated with the extent of chromatin stretching (Tajik et al., 2016). Importantly, the gene upregulation of DHFR (an essential molecule for the synthesis of thymine) is dependent on the surface stress angle relative to the cell long axis for the same magnitude of the stress and the initiation of gene activation is within milliseconds of load application, indicating that the gene is activated directly by chromatin stretching without the relaying assistance from the intermediate cytoplasmic biochemical signaling cascades (Tajik et al., 2016). It is also shown that BAF (barrier‐to‐autointegration factor) is an important structural protein that transmits the stress from the nuclear lamina to the chromatin since knocking down BAF inhibits external stress‐induced gene upregulation. Further studies reveal that endogenous genes egr‐1 (early growth response‐1) and Cav1 (calveolin‐1) are rapidly activated by a local stress via integrins and the stress‐induced gene activation depends on the chromatin domain being demethylated at histone 3 at lysine 9 (H3K9) (Sun, Chen, Mohagheghian, & Wang, 2020). Additionally, cytoskeletal prestress regulates the gene activation and upregulation induced by either a complex stress (both tensile stress and shear stress) or a shear stress on the cell surface via integrins (Wei et al., 2020), indicating control of anisotropic gene activation by the cytoskeletal prestress. All these findings suggest that cytoskeletal prestress plays a critical role in regulating chromatin stretching and rapid gene transcription (Figure 2).

The cytoskeletal prestress possibly regulates external stress‐induced strains on individual molecules and structures via controlling their modulus, stress focusing, stress propagation, molecular dynamics (on and off rates), and stress distribution as well as their cryptic sites. In other words, based on the current understanding of cellular responses, the critical parameter for the cell to respond may not be “the applied stress” per se. The “induced strain” or “induced deformation” in various components of the cell is likely the key regulator of cellular processes. It appears that the cell responds when the “induced strain” reaches a threshold. Since the induced strain depends on the applied stress divided by the modulus of the cell, which, in turn, depends on the cytoskeletal prestress, the induced strain is ultimately related to and depends on the cytoskeletal prestress. It is known that the cytoskeletal prestress ranges from ~100 Pa in cultured embryonic stem cells (Poh et al., 2009) to ~1,000 Pa in human airway smooth muscle cells (Wang et al., 2002). The applied stress by RGD‐coated magnetic beads via integrins ranges from ~5 to 20 Pa, which is in the same order of magnitude of shear stress (applied to the FAs of endothelial cells) induced by the blood flow from rest to exercise. Therefore, the magnitude of the applied stresses is only ~2–20% of the cytoskeletal prestress, high enough to trigger various cellular responses after reaching or exceeding threshold strains (>1–5%) for intracellular proteins, chromatins, and/or other structures (Johnson, Tang, Carag, Speicher, & Discher, 2007; Tajik et al., 2016). These stresses, applied for seconds to hours, do not appear too high or too long to cause cellular damage or apoptosis as the cells are able to differentiate (Chowdhury et al., 2009), contract, and/or proliferate long (hours to days) after stress application. It is important to note that cellular responses to applied stresses are known to be multifaceted and vary with time. Hence it is expected that the rapid gene transcription by force via direct chromatin stretching is followed by the slow processes of cytoplasmic mechanochemical signaling that depend on diffusion and/or translocation of cytoplasmic molecules into the nucleus. Together they elicit sustained cellular responses to externally applied stresses.

## 4 CYTOSKELETAL PRESTRESS IN EMBRYONIC DEVELOPMENT AND CELL FATE DETERMINATION

For decades the thrust of research on development is mainly focused on identifying genes responsible for development (Lewis, 1978; Nüsslein‐Volhard & Wieschaus, 1980). This line of research continues till today (Bronner, Feinberg, Roure, Piron, & Darras, 2019; Matsuo, Kuratani, Kimura, Takeda, & Aizawa, 1995; Niakan & Eggan, 2013; Reim, Frasch, & Schaub, 2017; Sozen et al., 2018) and is poised to reveal new findings for years to come (Gofflot, Jeannotte, & Rezsohazy, 2018). However, it is becoming increasingly evident that forces also help shape early embryonic development in addition to genetic control. During gastrulation, an early stage in development, cells undergo large deformation caused by patterned forces leading to major types of morphogenetic movements including invagination, ingression, involution, epiboly, intercalation, and convergent extension (Keller, 2002; Keller, Davidson, & Shook, 2003). Without these active movements, the germ‐layer formation is incomplete and subsequent development is halted. Compressive force has been shown to rescue the Twist protein expression and midgut formation in a mutant defective embryo in convergent‐extension movement (Desprat, Supatto, Pouille, Beaurepaire, & Farge, 2008; Farge, 2003; Pouille, Ahmadi, Brunet, & Farge, 2009). These convergent forces are shown to be essential in the developing embryos of *Drosophila*, *Xenopus*, and zebrafish (Breau et al., 2017; Dehapiot et al., 2020; Diaz de la Loza & Thompson, 2017; Keller & Danilchik, 1988; Keller & Shook, 2008; Kong, Wolf, & Großhans, 2017; LeGoff, Rouault, & Lecuit, 2013; Marsal, Hernández‐Vega, & Martin‐Blanco, 2017; Mongera, Michaut, Guillot, Xiong, & Pourquié, 2019; Shook, Kasprowicz, Davidson, & Keller, 2018; Shook & Keller, 2008; Sutherland, Keller, & Lesko, 2020; Yu & Fernandez‐Gonzalez, 2016; Zhou, Pal, Maiti, & Davidson, 2015). Recently a novel mechanotransduction pathway is discovered at tricellular junctions in the *Drosophila* embryo involving Abl tyrosine kinase and actin‐binding Canoe/Afadin that stabilizes cell adhesion under tension (Yu & Zallen, 2020).

In the early 2000s, several groups began investigating how cell shape affects cell fate decisions of adult stem cells (Kurpinski, Chu, Hashi, & Li, 2006; McBeath, Pirone, Nelson, Bhadriraju, & Chen, 2004). The effect of substrate stiffness on cell morphology and adhesion‐mediated cytoskeletal structures has been demonstrated (Yeung et al., 2005). Recently effects of viscoelasticity of the ECM on stem cell behaviors are also demonstrated (reviewed by Chaudhuri, Cooper‐White, Janmey, Mooney, & Shenoy, 2020). In various biological tissues from soft bone marrow to stiff bone, it is known that elastic (storage) modulus is in general ~10‐fold higher than dissipative (loss) modulus (Chaudhuri et al., 2020), suggesting that elastic stresses dominate the responses of living tissues and cells since the elastic modulus of the cells is also ~5–10‐fold higher than the dissipative modulus when the loading frequency is in the physiological range of 0.1–10 Hz. Despite the enormous advances in understanding cellular responses to substrate stiffness, the exact mechanism of how cells sense substrate stiffness remains unclear but a motor‐clutch model (Chan & Odde, 2008) and another molecular clutch model (Gong et al., 2018) seem to be able to explain the observed responses, although it often appears that some types of cells break the rule of the responses (Janmey, Fletcher, & Reinhart‐King, 2020).

A physical mechanism of jamming, non‐equilibrium phase transition from fluid phase to solid phase, has been proposed to explain changes in cell shape and geometry in monolayer epithelial cells (Park et al., 2015) and the *Drosophila* embryo (Atia et al., 2018). It is shown that the unjamming transition is distinct from the epithelial‐to‐mesenchymal transition in primary epithelial cells (Mitchel et al., 2020). Intracellular protein, RNA, and other biomolecules tend to form aggregates and condensates, especially for membraneless structures such as the nucleolus and the Cajal body, which is thought to be driven by the process of liquid–liquid phase separation (LLPS) (Shin & Brangwynne, 2017). The nucleolar size and shape depend on ATP (Brangwynne, Mitchison, & Hyman, 2011) and protein condensates in the nucleus of living cells depend on chromatin mechanics (Lee, Wingreen, & Brangwynne, 2021). On the other hand, an applied cell surface stress dissociates protein–protein complexes in the Cajal body that is critical for the biogenesis and recycling of small nuclear ribonucleoprotein complexes involved in pre‐mRNA splicing and pre‐ribosomal RNA processing (Poh et al., 2012). The propagation of the applied stress to the Cajal body depends on the cytoskeletal prestress. The Cajal body behaves as a solid‐like gel (Poh et al., 2012), although it may be formed via LLPS or other mechanisms. Compressive stresses initiate the transition from a solid‐like jammed phase to a fluid‐like unjammed phase, but the molecular mechanisms that underlie the jamming transition are not clear (Park et al., 2015). Recently it is shown that cadherins and ECM confinement cooperate to determine unjamming transitions and stepwise epithelial fluidization (Ilina et al., 2020). Currently, it is not clear if jamming/unjamming and LLPS describe the same or different phase transition process or are controlled by the same underlying physical mechanism. It also remains to be determined if and how LLPS processes in living cells are regulated by cytoskeletal and/or nuclear prestress.

It has been revealed that substrate elasticity directs human mesenchymal stem cell fate and the stem cell differentiation is blocked when cytoskeletal prestress is inhibited (Engler et al., 2006). Other studies have supported the notion that mechanical factors have a major impact on cell fate (Boontheekul, Kong, & Mooney, 2005; Dalby et al., 2007; Silva, Kim, Kong, & Mooney, 2008; Winer, Janmey, McCormick, & Funaki, 2009). Most of those early studies are carried out with multipotent adult stem cells. Nevertheless, how pluripotent stem cells would behave in response to force, whether endogenously generated or externally applied, had remained largely unknown in the early 2000s. It is then revealed that when the soft pluripotent mouse embryonic stem cells (mESCs) are subjected to external stress, they are more sensitive to stress magnitudes than differentiated tissue cells (Chowdhury et al., 2008; Chowdhury et al., 2009). Those mESCs generate low intrinsic cytoskeletal prestress and have very low intrinsic cell stiffness because of low levels of F‐actin (filamentous actin). With the application of a 20‐Pa external mechanical stress, mESCs start to spread, elevate their cytoskeletal prestress, exert elevated tractions on the underlying substrates, and eventually differentiate (Chowdhury et al., 2009). In contrast, when mESCs are cultured on soft substrates with a stiffness that mimic their intrinsic stiffness (~0.5 kPa), mESCs remain pluripotent without undergoing spontaneous differentiation in conventional culture (Chowdhury et al., 2010). The underlying mechanism for maintaining a homogenous self‐renewal of mESCs is attributed to the downregulation of cell‐matrix traction as a result of low cytoskeletal prestress on soft substrates that matches their intrinsic stiffness. When the tractions are elevated, the mESCs lose self‐renewal and pluripotency and begin to differentiate (Figure 2). This work has addressed a bottleneck problem of keeping homogenous self‐renewal of embryonic stem cells and preventing them from undergoing spontaneous differentiation in routine cell culture. Based on the understanding of substrate stiffness and cytoskeletal prestress on pluripotent stem cell differentiation, using a strategy of manipulating 3D matrix stiffness and matrix proteins, it is shown that a single mouse embryonic stem cell in culture can develop into a highly ordered and proper three‐germ layer arrangement of ecto‐, meso‐, and endoderm from the outer to the inner layer of the normal embryonic sphere (Poh et al., 2014). Using human pluripotent stem cells in mechanically‐designed cell culture environments, it is found that mechanics plays an important role in embryonic patterning that mimic early development in humans (Xue et al., 2018; Zheng et al., 2019). Next, we discuss the relationship between stem cells and tumor cells and the role of cytoskeletal prestress in tumorigenesis.

## 5 LOW PRESTRESS IN SOFT STEM CELL‐LIKE TUMOR CELLS PROMOTES TUMORIGENICITY AND METASTASIS

Human tumors are abnormal tissues that exhibit numerous unique hallmark traits (Hanahan & Weinberg, 2011). In the 1990s, it is demonstrated that not all cancer cells are the same and a few cancer cells in leukemia express stem cell surface markers and behave like stem cells and are thus called “cancer stem cells” (Bonnet & Dick, 1997; Lapidot et al., 1994). Following the report of cancer stem cells in leukemia, several additional studies have shown the existence of cancer stem cells in solid tumors: breast (Al‐Hajj, Wicha, Benito‐Hernandez, Morrison, & Clarke, 2003), brain (Singh et al., 2003), skin (Fang et al., 2005), prostate (Collins, Berry, Hyde, Stower, & Maitland, 2005), ovary (Bapat, Mali, Koppikar, & Kurrey, 2005), and lung (Eramo et al., 2008). However, whether cancer stem cells exist in solid tumors has been rather controversial (Ailles & Weissman, 2007; Quintana et al., 2008; Visvader & Lindeman, 2008), partly because some tumor cells are still able to initiate tumors without expressing the surface stem cell markers. A further study of human colon cancer finds that it harbors a tiny tumorigenic subpopulation that is uncorrelated with stem cell markers (Dieter et al., 2011), further questioning the existence of cancer stem cells in solid tumors. As a result, two different models of cancer are proposed: the “clonal evolution model” and the “cancer stem cell model”. In the clonal evolution model, it is believed that there exists a subpopulation of cells that carries advantageous mutations that allows them to grow even in the harshest condition. In contrast, the cancer stem cell model suggests that there is one stem cell during the onset of the tumor that is responsible for establishing the hierarchy in tumor organization that generates the entire diverse cell population. The discussion of these two models is highlighted in a review article (Shackleton, Quintana, Fearon, & Morrison, 2009). However, there is evidence that plasticity exists between the cancer stem cells and the differentiated noncancer stem cells and the noncancer stem cells can reacquire a cancer stem cell phenotype (Chaffer, Weinberg, & Marjanovic, 2013). Together these findings suggest that factors other than surface stem cell markers regulate tumorigenicity of cancer cells.

Based on the fact that self‐renewing stem cells are much softer and generate lower cytoskeletal prestress than differentiated progenies, it is hypothesized that a soft microenvironment would facilitate self‐renewal and selection of soft tumorigenic cancer cells. It is found that soft 3D fibrin gels with stiffness ~90 Pa allow the selection and proliferation of highly tumorigenic cells from a general population of tumor cells in culture (Liu et al., 2012). As the stiffness of the 3D fibrin gels is increased, the spheroid‐forming efficiency, as well as tumorigenicity of the cells, decreases dramatically, signifying the importance of the soft microenvironment in harboring these tumorigenic cells. These highly tumorigenic and metastatic tumor cells, called tumor repopulating cells, because they appear to be distinct from conventional surface stem cell marker selected cancer stem cells or tumor‐initiating cells, express similar levels of cancer stem marker CD133 as the parental melanoma cells but are >100‐fold more efficient in generating tumors and melanoma metastasis in the lung of wild‐type mice (Liu et al., 2012). These melanoma tumor‐repopulating cells express high levels of self‐renewing gene *Sox2* and exhibit a very low intrinsic cell stiffness of ~0.5 kPa (Tan et al., 2014) (Figure 2). Interestingly, when these cells are plated on a *2D* substrate of stiffness ranging from ~0.1 to 8 kPa to rigid plastic dish, they maintain their low cell stiffness of 0.5 kPa and thus low cytoskeletal prestress for at least 24 hr (Tan et al., 2014), quite different from the stiffening behaviors observed from normal tissue cells in response to elevating substrate stiffness (Tee, Fu, Chen, & Janmey, 2011). The epigenetic modifications like hypermethylation or demethylation of H3K9 alter the expression of *Sox2*, changing the self‐renewing capabilities of these tumorigenic tumor‐repopulating cells (Tan et al., 2014). A zebrafish model using different fluorescence markers to label tumor‐repopulating cells and the un‐selected differentiated tumor cells reveals that low cytoskeletal prestress and low F‐actin are the key determinants in promoting extravasation efficiency of these soft tumor cells whereas increasing F‐actin inhibits extravasation (Chen et al., 2016), consistent with the notion that soft and low prestress tumor‐repopulating cells are able to penetrate blood vessels more easily to extravasate than their stiff counterparts. The detailed regulatory processes of tumor cell intravasation and extravasation in vivo, however, are still not well understood (Kai, Drain, & Weaver, 2019). As such, in vitro culture models are built to simulate these processes. 3D microfluidic models have been applied to study intravasation or extravasation and it is shown that endothelial barrier impairment is associated with a high number and fast dynamics of tumor cell‐endothelial cell interactions during intravasation (Zervantonakis et al., 2012) and monocytes directly reduces cancer cell extravasation (Boussommier‐Calleja et al., 2019). It is also shown that platelet decoys decrease tumor cell extravasation in a microfluidic model and inhibit thrombosis in rabbits and prevent metastatic tumor formation in mice (Papa et al., 2019). To identify the biomarkers of the melanoma tumorigenic cells, next‐generation sequencing‐based RNA sequencing approach was adopted. The RNA sequencing data have identified differentially expressed cell adhesion cluster in these soft melanoma tumor‐repopulating cells, which leads to the identification of highly specific and novel biomarkers like Col2a1, Ncam1, F11r, and Negr1 (Talluri et al., 2020). Similarly, transcriptome analysis of soft tumor‐repopulating cells of human cervical cancer Hela cell line reveals *CCT3* as a putative stemness‐related gene (Huang et al., 2019).

Cancer stem cells from solid tumors appear to share the capacity of self‐renewal with normal stem cells (Bapat, 2010) but have a distinct feature of metastasis that normal stem cells do not have. An early study reveals spontaneous and transplantable testicular teratoma in mice (Stevens & Little, 1954), suggesting the existence of “abnormal stem cells” in the teratoma. At the level of DNA, it is proposed that the formation of critically short telomeres in cancer stem cells, distinct from normal stem cells, instigates genomic instability and initiation of breast cancer stem cells with metastatic potential (Robinson, Taylor, & Schiemann, 2019). In contrast, telomere shortening or attrition is found to cause cell cycle arrest in human induced pluripotent stem cells–derived cardiomyocytes (hiPS‐CMs) and osteosarcoma cells (Cho et al., 2019). However, it is not clear at this time if a few of these normal hiPS‐CMs can come out of cell cycle arrest and progress to become cancer stem cells or if some cells in the general population of osteosarcoma cells behave like cancer stem cells and thus do not undergo cell cycle arrest. Stem‐cell‐like tumor‐repopulating cells, like the normal stem cells, are also capable of self‐renewal (J. Liu et al., 2012; Tan et al., 2014). In contrast, tumor‐repopulating cells, like cancer stem cells derived from solid tumors, are highly metastatic. Future studies are needed to determine if tumor‐repopulating cells differ from normal stem cells in terms of telomere shortening and/or other mechanisms at the genetic level.

Tumorigenic cells can be characterized by their softness which can be potentially used as an inherent biomarker. An optical stretcher has been developed to measure optical deformability of suspended metastatic versus normal breast epithelial cells and found that the metastatic cells are softer and deforms more than normal cells (Guck et al., 2005). AFM measurement of fresh intact biopsy breast cancer tissue samples shows that the metastatic cell population contains three subpopulations with one population being very soft, most likely to be responsible for metastasis (Plodinec et al., 2012). An ex vivo analysis of cells from metastatic cancer patient samples using AFM also shows a low stiffness profile (Cross, Jin, Rao, & Gimzewski, 2007). A recent report that utilizes the softness trait to separate soft tumor cells from stiff tumor cell population using a microfluidic‐based method reveals that these soft cells are much more tumorigenic and metastatic than stiff tumor cells (Lv et al., 2020), suggesting that the cell softness is a physical marker for malignant solid tumors (Figure 2), adding a new physical trait to the known four physical traits (tumor tissue solid stress, interstitial fluid pressure, tumor tissue stiffness, and tumor microarchitecture) of solid tumors that may hamper successful treatment of malignant tumors (Nia, Munn, & Jain, 2020). The stiffness of these malignant soft tumor cells in suspension is only one‐third of the stiffness of those differentiated tumor cells (Lv et al., 2020), suggesting that their intrinsic cytoskeletal prestress is much lower than that in those stiff tumor cells. It is generally known that some tumors such as breast cancer are stiff and can be felt by palpation. There is evidence that tension‐dependent matrix stiffening facilitates breast cancer progression (Levental et al., 2009; Paszek et al., 2005; Samuel et al., 2011) and glioblastoma invasion (Barnes, Przybyla, & Weaver, 2017). How would one reconcile the findings of the stiff tumor tissues with those of the soft malignant stem cell‐like tumor‐repopulating cells in cancer progression? It is likely that tumor tissues, although stiffened due to elevated matrix tension or excess collagen deposition, are heterogeneous in their stiffness. It is this tumor tissue stiffness heterogeneity that potentiates tumor cell differentiation and invasion. Those undifferentiated tumor cells, such as the soft tumor‐repopulating cells, follow those differentiated tumor cells to invade and to intravasate. It is those soft undifferentiated or partially differentiated tumor cells that are likely the culprit in establishing metastatic colonization (Tan et al., 2014). A recent study (Jiang et al., 2020) shows that lowering tissue stiffness in pancreatic ductal adenocarcinoma metastasis in the liver accelerates tumor growth and results in diminished overall survival, suggesting the stiffening of the tumor tissue is not the culprit for tumor growth at the metastatic sites. Another recent study demonstrates that differential tissue stiffness is the key to triggering an invasion of skin tumor stem cells in a developing embryo (Fiore et al., 2020). Additional evidence of uniform stiff matrix limiting tumor progression comes from the finding that a homogenous 3D stiff matrix triggers dormancy of stem‐cell‐like soft tumor repopulating cells via a Cdc42‐driven Tet2 epigenetic process in mouse models for both murine and primary human melanoma (Y. Liu et al., 2018). A report on breast cancer cells in culture finds that these cells entering dormancy form a fibrillar fibronectin matrix and exits from dormancy require MMP‐2‐mediated fibronectin degradation (Barney et al., 2020), suggesting that stiff fibronectin matrices surrounding the tumor cells promote breast cancer dormancy. All these findings paint a picture of stiffness matching (Chen & Wang, 2018): intrinsically soft stem‐cell‐like tumor cells that have low prestress thrive in a soft 3D matrix environment whereas stiff differentiated tumor cells benefit in a stiff 3D matrix environment. The evidence that a uniform stiff ECM hinders tumor growth is consistent with the proposition that the ECM is a (physical) barrier to restrain tumor progression (Bissell, 1981; Bissell & Hines, 2011). We propose that stiffening of the tumor tissue microenvironment is a protective response of the body trying to contain the abnormal tissue growth, leading to tumor dormancy if successful and to tumor invasion/metastasis if unsuccessful. The model of the soft tumor cells such as stem cell‐like tumor‐repopulating cells that are undifferentiated or partially differentiated being the primary culprit of tumor metastasis needs to be tested in the future.

## 6 CYTOSKELETAL PRESTRESS IN IMMUNE CELLS

Since it is well‐established that endogenous cytoskeletal forces play important roles in developing embryos, pluripotent stem cells, adult stem cells, and differentiated cells, it is logical to deduce that forces are also at play in immune cells since these cells are all generated from bone marrow stem cells. Actually, it has been proposed a long time ago that all cells respond to mechanical stresses (Davies & Tripathi, 1993). The immune system functions as the host defense against infection and includes innate and adaptive systems. The nonspecific innate system is made of complement components and innate immune cells (macrophages, dendritic cells, granulocytes, NK [natural killer] cells, and mast cells) while the adaptive immune system is specific and is made of T cells and B cells. Neutrophil deformation is measured after the cell is sucked into a micropipette (Evans & Kukan, 1984). Later a sensitive micropipette‐based piconewton (pN) (1 pN = 10^−12^ Newton) force transducer to quantify neutrophil membrane stiffness dynamics has been developed (Simon et al., 2007). Adhering to TNFα (tumor necrosis factor‐alpha)‐activated endothelial cells, neutrophils elevate their stiffness within 2 min (Wang et al., 2001). It is known that neutrophils migrating on compliant substrates generate tractions (Jannat, Dembo, & Hammer, 2011). Neutrophils are also known to exert tractions during the process of diapedesis and actively contract the vascular endothelial cells to open a junctional gap and then push themselves across the gap (Yeh et al., 2018). A recent study using a FRET sensor shows direct evidence of leukocytes generating tension on VE‐cadherin during trans‐endothelial migration (Arif et al., 2021). Like neutrophils, monocytes are also able to cross the endothelium, entering the tissue parenchyma where they readily differentiate into macrophages. As professional phagocytes, macrophages phagocytize varieties of foreign materials including apoptotic or senescent cells to maintain the human body's homeostasis. Notably, this phagocytosis is regulated by the target cell's stiffness and the cytoskeletal contractile prestress (Alvey et al., 2017; Andrechak, Dooling, & Discher, 2019; Subramanian, Parthasarathy, Sen, Boder, & Discher, 2006). Moreover, the trigger of phagocytosis necessitates macrophage membrane distortion and deformation. In line with this biological function, macrophages are indeed soft. Since activated macrophages can be polarized toward M1 or M2 phenotype, whether and how the prestress regulates the macrophage‐polarizing process is intriguing and worthy of investigation. Compared with macrophages or other innate immune cells, adaptive immune cells are much smaller. However, their priming and activation are also dependent on cytoskeletal prestress. Over the last several years it is shown that contractile forces regulate T cell activation (Agrewala et al., 2011; Basu et al., 2016; Kellermayer, Hong, Murugesan, Betzig, & Hammer, 2017) (reviewed by (Blumenthal & Burkhardt, 2020) (Figure 2). It has been shown that cytotoxic T cells use their cytoskeletal contractile prestress to kill tumor cells (Basu et al., 2016). However, how tumor cells in turn use their own contractile prestress to counteract T cell killing has been unclear. It is only recently revealed that malignant tumor‐repopulating cells evade cytotoxic T cell killing through a mechanical softness mechanism by impairing perforin pore formation. Downregulating this softness of tumor cells (i.e., elevating tumor cell stiffness) restores T cell‐mediated cytolysis of tumor‐repopulating cells (Liu et al., 2021). This killing process occurs at the immune synapse site where CD8^+^ T cells contact with tumor cells and release perforin and granzymes to mediate the killing. The space within the immune synapse is relatively sealed off and the perforin not only acts on tumor cells but should also attack T cells; how T cells evade this “self” killing is not well understood. Together these published reports highlight the importance of cytoskeletal prestress in immune cells and in target cells in generating effective immune responses (Figure 2).

## 7 EXTRACELLULAR VESICLES AND PRESTRESS

Soluble factors like growth factors and cytokines, in addition to autocrine and paracrine signaling, depend on diffusion and fluid flows to impact their targets far away from the source. Because of the dilution of the molecules, it is difficult to have a sustained impact on cells and tissues. On the other hand, the physical forces can only be exerted via cell‐matrix or cell–cell contacts. Over the last decade, another form of signaling has been discovered by the cells: extracellular vesicles (EVs), a structure with a size between that of molecules and cells (Colombo, Raposo, & Théry, 2014). This type of signaling is a signaling that is more sustained than soluble factor‐induced signaling but more far‐reaching (via fluid flows) than force‐induced signaling that requires physical contact. Two types of EVs have been identified: exosomes and microparticles (MPs). Exosomes are generated in multivesicular bodies with small sizes (30–100 nm); they are released from the endosomes upon fusion with the plasma membrane to the extracellular space (Raposo & Stoorvogel, 2013). Such endosome‐derived EVs deliver proteins, mRNAs, and microRNAs to recipient cells. MPs are plasma membrane‐derived shedding vesicles with sizes ranging from 0.1 to 1 μm (Chen et al., 2019). In response to various stimuli, upon the release of Ca^2+^ from the endoplasmic reticulum, cells change their cytoskeletal structure and lead to the encapsulation of cytosolic components by the plasma membrane, followed by the release of vesicles into the extracellular space. In some studies, MPs are also known as microvesicles (MVs). Both exosome and MP releases are regulated by cytoskeletal movements and structure changes and the cellular prestress are involved in this process. Ultraviolet irradiation can induce tumor cells to release abundant MPs; however, this releasing is blocked by either cytochalasin D, an inhibitor of F‐actin polymerization, or blebbistatin, an inhibitor of myosin‐II mediated actin filament motility (Tang et al., 2012). Tumor cells not only release MPs but also can take up MPs. Intriguingly, treatment with cytochalasin D or blebbistatin may enhance the uptake of MPs by tumor cells. Thus, myosin‐II dependent cytoskeletal prestress might mediate the release of MPs by tumor cells, whereas a soft microfilament cytoskeleton is perhaps more suitable for MP uptake (Figure 2). Following the uptake, cargoes (MPs) are delivered from endosomes to lysosomes where acidic enzymes‐mediated degradation may occur and this delivery is undoubtedly regulated by cytoskeletal prestress and cytoskeletal movements. Based on such biomechanical understanding, a tumor cell‐derived, drug‐loaded MP treatment platform has been developed. Highly tumorigenic tumor‐repopulating cells are very soft (Lv et al., 2020) and preferable to take up drug‐packing MPs via their high deformability. In turn, MPs mobilize the endo‐lysosomal systems, allowing the drug molecules to be delivered to the nucleus, thus killing tumor‐repopulating cells and reversing their drug resistance (Jin et al., 2017; Ma et al., 2016). Moreover, the softness of tumor‐repopulating cells can be further exploited in cancer treatment. Tumor repopulating cell‐generated MPs are softer than their counterparts generated from differentiated tumor cells. Such softness results in an enhanced anti‐cancer drug‐MP accumulation in tumor tissues, an enhanced blood‐vessel crossing and penetration into tumor parenchyma, and a preferential uptake by tumor‐repopulating cells and thus their killing by the drug‐MPs (Liang et al., 2019) (Figure 2). It is increasingly evident that the cytoskeletal prestress regulates growth factor‐induced signaling and responses and vice versa. In the future, cross‐talks, regulation, and feedback loops among these forms of signaling‐soluble molecules, exosomes and microparticles, stresses (cytoskeletal prestress and external stress), mechanical cues of the microenvironment, together with neural cell‐generated (action potential dependent) electrical signaling, need to be carefully investigated to get a deeper understanding of responses to stimuli and functions of cells and tissues in the body.

## 8 SINGLE‐MOLECULE FORCE SENSATION IN A LIVING CELL

To understand the underlying mechanism of how single cells respond to an applied force, molecular force probes are necessary. Single‐molecule techniques have been applied to study bacteria and biological molecules for three decades (Block, Blair, & Berg, 1989; Block, Goldstein, & Schnapp, 1990; Ha et al., 1999; Hansma, Elings, Marti, & Bracker, 1988; Liphardt, Onoa, Smith, Tinoco Jr, & Bustamante, 2001) but their applications to living animal cells are realized only recently. It is important to note that single‐molecule techniques need to be custom‐tailored to specifically address the research questions involving living animal cells. In 2010, A FRET‐based genetic biosensor allowing force measurements across vinculin proteins in living cells with piconewton (pN) sensitivity has been developed and calibrated (Grashoff et al., 2010). Following this report, several studies developed FRET‐based genetic biosensors for measuring forces across different proteins including E‐cadherin, VE‐Cadherin, and PECAM‐1 (Austen et al., 2015; Borghi et al., 2012; Cai et al., 2014; Chang et al., 2016; Conway et al., 2013; LaCroix, Lynch, Berginski, & Hoffman, 2018). It is shown that a large fraction (60–80%) of integrins bear very modest loads of 1–3 pN where only a small fraction (<10%) of integrins bear loads of >7 pN (Chang et al., 2016), which may partially explain why early force values from single‐molecule force measurements on a single integrin are several orders of magnitude higher than those average force values on integrins from traction measurements at a single cell level. However, how forces are distributed over different fractions of integrins in a single cell remain unclear at this time. Several groups adopted another approach to developing DNA hairpin‐based FRET probes to quantify traction forces (Blakely et al., 2014; Zhang, Ge, Zhu, & Salaita, 2014). A fluorescence biomembrane force probe (fBFP) is used to study T cell activation and it is revealed that T cell receptor (TCR) and peptide‐major histocompatibility complex (pMHC) form “catch bonds” (Liu, Chen, Evavold, & Zhu, 2014). It is also shown that frequently applied forces on TCR and CD8 trigger calcium entry (from both intracellular and extracellular sources) into the cytoplasm of T cells and that stiffened antigen presenting cells enhance the calcium response (Pryshchep, Zarnitsyna, Hong, Evavold, & Zhu, 2014). As force is a vector quantity, measuring or altering only magnitude but not direction becomes a limiting factor for these methods. Recently, the Salaita group addressed this issue of measuring both magnitude and directions of integrin traction forces with piconewton resolution (Brockman et al., 2017). Piconewton force measurements have been performed for mapping cell–cell adhesion molecules (e.g., E‐cadherin) (Chang, Liu, et al., 2016) and even for measuring forces during growth factor receptor activation, which depends on the integrity of F‐actin (Stabley, Jurchenko, Marshall, & Salaita, 2011), suggesting the dependence on cell cortex tension (cortex cytoskeletal prestress). One caveat of these FRET probes may arise from the signal quenching effect by the cells, particularly in the case of weak FRET signals.

A different approach was developed, called tension gauge tethers (TGTs), to measure molecular forces within 10–60 pN range (Wang et al., 2015, 2016; Wang & Ha, 2013). These studies suggest cells cannot apply more than 40 pN peak molecular force via single integrins. Using the TGT platform, the molecular force‐dependent cell spreading mechanism was also revealed (Chowdhury et al., 2015). Using TGT to study forces generated by tumor cells, it is found that highly tumorigenic tumor‐repopulating cells do not exhibit molecular force‐dependent cell spreading behavior due to severe downregulation of *Cdc42* (Chowdhury et al., 2018). Using improved tension probes with low tension tolerance, it is revealed that Notch is activated by force (Chowdhury et al., 2016; Gordon et al., 2015).

One major challenge in any single‐molecule probes is force calibration. While some studies assign an arbitrary loading rate or report a range of loading rates during the rupture force calibration, others do not report any loading rate for rupture force calibration measurements. The loading rate is crucial for determining ligand‐receptor bond characteristics. Therefore, it is imperative to know the range of physiologically relevant cellular loading rates. However, measuring physiologically relevant loading rates can be a daunting task (Maruthamuthu, Schulten, & Leckband, 2009). A recent study has demonstrated the feasibility of determining cellular loading rate (80–115 pN/s) during early cell adhesion events (Amar, Suni, & Chowdhury, 2020). Future investigations are necessary to evaluate the dependence of cellular loading rates on ligand‐receptor types, cell location within the tissue, and cytoskeletal prestress.

## 9 OUTLOOK FOR MECHANOBIOLOGY AND MECHANOMEDICINE

The application of biomechanics to medicine and health has existed for decades. Over the last few decades, biomechanics has reached a mature stage and a significant amount of the relevant research currently performed in the field deals with achieving predictive, multi‐scale, and integrative models accounting for different phenomena (e.g., fluid–structure interactions, tissue mechanics, growth and remodeling, and electrophysiology in the heart). With the ever‐increasing amount of information at the microstructural level (e.g., the ECM and the cellular level), the challenges faced by the field of biomechanics are still substantial. On the other hand, mechanobiology, which may be considered as a branch of biomechanics and/or a branch of biology, has been emerging mainly because of the significant technological and methodological advances at the cellular, subcellular, and molecular levels and the need to unravel the mechanical underpinnings of biology. Mechanobiology has benefited greatly from advances in biomaterials (Abar et al., 2020; Lendlein & Langer, 2002; Wang et al., 2020; Yang, Tibbitt, Basta, & Anseth, 2014), nanotechnologies (Hirsch et al., 2003; Korin et al., 2012; Mitchell et al., 2021; Norman & Desai, 2006; O'Neal, Hirsch, Halas, Payne, & West, 2004; Peer et al., 2007), soft lithography (Kane, Takayama, Ostuni, Ingber, & Whitesides, 1999), microdevice fabrication to create organs on a chip (Huh et al., 2010), molecular engineering of fluorescent probes (Wang, Shyy, & Chien, 2008), stem cell technologies (Brons et al., 2007; Magnuson, Epstein, Silver, & Martin, 1982; Shi, Inoue, Wu, & Yamanaka, 2016; Takahashi & Yamanaka, 2006; Tesar et al., 2007; Thomson et al., 1998), and tissue engineering (Lanza, Langer, Vacanti, & Atala, 2020). Over the last decade, some promising advances in mechanomedicine have been made in drug testing/delivery and cancer diagnosis/treatment (Ali, Emerich, Dranoff, & Mooney, 2009; Chen et al., 2019; Herland et al., 2020; Jain, 2013; Jalil, Andrechak, & Discher, 2020; Kantamneni et al., 2017; Liang et al., 2019; Ma et al., 2016; Mpekris et al., 2020; Shen et al., 2020). Among them, a couple of studies have employed the strategy of modulating prestress for effective drug delivery using soft‐tumor‐cell‐derived microparticles (Liang et al., 2019; Ma et al., 2016). Based on the knowledge on myosin and its interaction with actin, molecules targeting myosin and its associated proteins have been utilized in clinical trials for prevention and early treatment of cardiomyopathies (Repetti, Toepfer, Seidman, & Seidman, 2019). However, approaches for treating patients using mechanobiology‐derived strategies are just emerging. Some examples of how mechanobiology‐based approaches have shaped clinical responses and outcomes are (a) a therapeutic, biomaterial‐based, cancer vaccine technology (Ali et al., 2009) that has started clinical trials in 2013 and has been commercialized by Novartis in 2018; (b) a tumor‐cell‐derived chemotherapeutic microparticle technology that reverses cancer drug resistance in terminal stage‐IV cancer patients (Ma et al., 2016) has entered clinical trials with promising results of extending survival rates of stage‐IV cancer patients; (c) Shear stress‐activated nanotherapeutics technology for treating thrombosis (Korin et al., 2012) is also in clinical trials.

However, major challenges in the application of mechanobiology to mechanomedicine continue to persist. In living tissues and living animals, it is more daunting and challenging than in cultured cells to apply, identify, and manipulate mechanical inputs and signals since it is much harder to separate mechanical signaling and mechanisms from soluble biochemical signals and electrical signals in vivo than in vitro. For example, it is rather challenging to quantify tissue stiffness and tractions and to estimate cytoskeletal prestress in cells in a human body or a living animal in physiology or disease. In addition, it is difficult to locally modulate mechanical properties and cytoskeletal prestress of an individual cell or a group of cells without changing nearby cells or their microenvironment in a human body. One issue involves the size and the delivery mode of the mechanobiology‐based devices, gadgets, or drugs that can be exploited to alter the clinical outcomes of the patients that traditional approaches cannot, although a few successful applications in mechanomedicine have been achieved so far (see above). Therefore outside‐the‐box thinking is needed to develop novel ways to diagnose and treat patients using mechanobiology‐based strategies. It is imperative that mechanobiology‐based approaches and technologies be combined with genetically (e.g., CRISPR and others) and soluble‐factor based manipulations and novel whole‐body imaging modalities to improve diagnostics, treatment, and therapeutics in medicine. These challenges wait for scientists and researchers to meet by working together to develop new approaches and to identify novel pathways to intervene. Nevertheless, the next few decades should be exciting for scientists working in the field of mechanobiology to advance mechanomedicine to make an impact in medicine and health.

## CONFLICT OF INTEREST

The authors declare no conflict of interest.

## Data Availability

Data sharing is not applicable to this article as no new data were created or analyzed in this study.
